# Characterization of the SARS-CoV-2 B.1.621 (Mu) variant

**DOI:** 10.1126/scitranslmed.abm4908

**Published:** 2022-05-17

**Authors:** Peter J. Halfmann, Makoto Kuroda, Tammy Armbrust, James Theiler, Ariane Balaram, Gage K. Moreno, Molly A. Accola, Kiyoko Iwatsuki-Horimoto, Riccardo Valdez, Emily Stoneman, Katarina Braun, Seiya Yamayoshi, Elizabeth Somsen, John J. Baczenas, Keiko Mitamura, Masao Hagihara, Eisuke Adachi, Michiko Koga, Matthew McLaughlin, William Rehrauer, Masaki Imai, Shinya Yamamoto, Takeya Tsutsumi, Makoto Saito, Thomas C. Friedrich, Shelby L. O’Connor, David H. O’Connor, Aubree Gordon, Bette Korber, Yoshihiro Kawaoka

**Affiliations:** ^1^ Department of Pathobiological Sciences, School of Veterinary Medicine, University of Wisconsin, Madison, WI 53711.; ^2^ Los Alamos National Laboratory, Space Data Science and Systems, Los Alamos, NM 87545.; ^3^ Department of Pathology and Laboratory Medicine, University of Wisconsin, Madison, WI 53705.; ^4^ UW Health Clinical Laboratories, University of Wisconsin Hospital and Clinics, Madison, WI 53792.; ^5^ Division of Virology, Department of Microbiology and Immunology, Institute of Medical Science, University of Tokyo, 108-8639 Tokyo, Japan.; ^6^ Department of Pathology, University of Michigan, Ann Arbor, MI 48109.; ^7^ Department of Internal Medicine, Division of Infectious Diseases, University of Michigan, Ann Arbor, MI 48109.; ^8^ Wisconsin National Primate Research Center, University of Wisconsin, Madison, WI 53715.; ^9^ Division of Infection Control, Eiju General Hospital, 110-8645, Tokyo, Japan; ^10^ Department of Hematology, Eiju General Hospital, 110-8645, Tokyo, Japan; ^11^ Department of Infectious Diseases and Applied Immunology, IMSUT Hospital of The Institute of Medical Science, University of Tokyo, 108-8639, Tokyo, Japan; ^12^ Division of Infectious Diseases, Advanced Clinical Research Center, Institute of Medical Science, University of Tokyo, 108-8639, Tokyo, Japan; ^13^ Department of Epidemiology, School of Public Health, University of Michigan, Ann Arbor, MI 48109.; ^14^ Los Alamos National Laboratory, Theoretical Biology and Biophysics, Los Alamos, NM 87545.; ^15^ The New Mexico Consortium, Los Alamos, NM 87545.; ^16^ The Research Center for Global Viral Diseases, National Center for Global Health and Medicine Research Institute, Tokyo 162-8655, Japan.

## Abstract

The SARS-CoV-2 B.1.621 (Mu) variant emerged in January 2021 and was categorized as a variant of interest by the World Health Organization in August 2021. This designation prompted us to study the sensitivity of this variant to antibody neutralization. In a live virus neutralization assay with serum samples from individuals vaccinated with the Pfizer/BioNTech or Moderna mRNA vaccines, we measured neutralization antibody titers against B.1.621, an early isolate (spike 614D), and a variant of concern (B.1.351, beta variant). We observed reduced neutralizing antibody titers against the B.1.621 variant (3.4 to 7-fold reduction, depending on the serum sample and time after the second vaccination) compared to the early isolate and a similar reduction when compared to B.1.351. Likewise, convalescent serum from hamsters previously infected with an early isolate neutralized B.1.621 to a lower degree. Despite this antibody titer reduction, hamsters could not be efficiently re-challenged with the B.1.621 variant, suggesting the immune response to the first infection is adequate to provide protection against a subsequent infection with the B.1.621 variant.

## Teaser

Immunity acquired from a previous infection or vaccination limits replication of the B.1.621 (Mu) variant.

## INTRODUCTION

Variants of the original severe acute respiratory syndrome coronavirus 2 (SARS-CoV-2) strain that emerged in 2019 have continued to evolve as mutations accumulate throughout the viral genome, including in the gene encoding the spike protein. As the virus adapts in humans and evolves to evade the immune system, the mutations observed in the variant viruses have the potential to alter transmission and disease, as well as the susceptibility to antibody responses elicited against previous infection or vaccination. An emerging variant can be classified as a variant of interest, variant of concern, or variant of high consequence; these designations are based on prevalence and how great an impact the amino acid substitutions within the viral proteins in the variant have on transmission and key countermeasures such as vaccines, therapeutics, and diagnostics. The World Health Organization (WHO) has designated B.1.1.7 (Alpha), B.1.351 (Beta), P.1 (Gamma), and B.1.617.2 (Delta) as variants of concern. Recently, lineages in B.1.529 (Omicron; BA.1, BA.1.1, and BA.2) have dominated the SARS-CoV-2 landscape as variants of concern.

Here, we characterized the variant B.1.621 (Mu), which spread worldwide in 2021 with high prevalence in North and South America. With B.1.621 in a live virus assay, we observed a reduction in neutralizing antibody titers in serum from individuals vaccinated with mRNA vaccines. However, despite this reduction, in the hamster model of infection, B.1.621 was unable to establish infection in the lower respiratory tissues of previously infected animals.

## RESULTS

### Prevalence of the B.1.621 variant

On August 31, 2021, the family of SARS-CoV-2 genotypes that includes the Pango lineages B.1.621 and B.1.621.1 was designated as a WHO variant of interest, called Mu (*1*). This variant was first detected in January 2021 in Columbia (accession number: EPI_ISL_1220045) and has since spread throughout the Americas (fig. S1) and into Europe (*2*, *3*). As of September 2021, 5,327 sequences of B.1.621 or B.1.621.1 have been sampled in 39 countries (based on the Global Initiative on Sharing Avian Influenza Data [GISAID] database). The B.1.621 variant had an increasing presence in some regions in the Caribbean, United States (fig. S2), and South America over the summer of 2021 prior to the B.1.617.2 (Delta) variant surge, whereas in Chile and Colombia, the B.1.621 variant was the most commonly sampled lineage of SARS-CoV-2 at that time (fig. S1) (*4*). In addition, a subvariant of B.1.621 containing a K417N substitution in the spike protein emerged in the United States, Mexico, and the United Kingdom (fig. S3). Ultimately though, the B.1.621 variant eventually disappeared. By June 2021, the B.1.617.2 variant dominated and replaced B.1.621. This global surge of B.1.617.2 was potentially due to its higher transmissibility relative to B.1.621.

### Isolation of a B.1.621 virus and characterization in mice

In this study, we isolated a B.1.621 variant from a nasopharyngeal (NP) swab from a healthcare worker as part of contact tracing during an outbreak investigation. The individual tested positive for SARS-CoV-2 approximately 13 weeks after receiving the second dose of the Pfizer vaccine. Virus from the NP swab was isolated and passaged once on Vero E6-transmembrane serine protease 2 (TMPRSS2) cells (*5*). The sequence of the virus in the swab and the first passage of virus were identical and classified as the SARS-CoV-2 variant B.1.621 (GISAID accession ID: EPI_ISL_1827552; hCoV-19/USA/WI-UW-4340/2021). A comparison of the B.1.621 genome to a reference genome (Wuhan-Hu-1; GenBank ID: MN908947) revealed ten amino acid substitutions and one amino acid insertion in the spike protein of B.1.621: a substitution in the N-terminal domain (NTD; T95I), together with two substitutions and an insertion at positions 144 to 145 (YY to TSN) in the NTD; three substitutions in the receptor-binding domain/motif (R346K, E484K, N501Y); the D614G substitution; one substitution proximal to the furin cleavage site (P681H); and two substitutions in the S2 region (D950N, N1074K).

These substitutions may reduce antibody recognition of the spike protein, thus potentially reducing antibody neutralization of the virus by convalescent serum or serum from vaccinated individuals. Specifically, the altered amino acid sequence in the NTD of B.1.621 may affect neutralization by this type of antibody, given that there have been reports of antibody resistance with the B.1.1.7 (Alpha) variant and other lineages with the 144Y deletion and that NTD neutralizing antibodies make contact at amino acid positions 144 and 145 (*6*, *7*). The R346K mutation, categorized as a Barnes class III-type antibody site, and the E484K mutation, categorized as a Barnes class II-type antibody site, may lead to reduced antibody recognition of the receptor-binding domain of the B.1.621 spike protein (*8*). In addition, the E484K mutation has been shown to affect antibody resistance to convalescent and vaccine serum samples (*9*, *10*). The spike protein of B.1.621 also possess an N501Y mutation, which may impact antigenicity and binding to human angiotensin converting enzyme 2 (ACE2) (*11*, *12*). The N501Y mutation also enhances binding to mouse ACE2, enabling virus replication in mice (*13*, *14*).

To confirm the ability of B.1.621 to infect and replicate efficiently in mice, we infected C57BL/6J mice (males; 12-weeks old; n=4) with 10^4^ plaque-forming units (pfu) of B.1.621 by intranasal inoculation. On day 3 after infection, B.1.621 replicated to high virus titers in the lungs of all animals (fig. S4). By day 6 post-infection, virus titers had declined (fig. S4). Although no outward clinical signs of infection were observed, including no weight loss, the ability of this variant to replicate efficiently in mice makes mice a useful animal model to evaluate vaccines and therapeutics against B.1.621.

### Neutralization of B.1.621 by serum from vaccinated individuals

We have scant data about the sensitivity of B.1.621 to neutralizing antibodies in serum from individuals vaccinated twice with one of the available mRNA vaccines. Therefore, to determine the sensitivity of B.1.621 to neutralizing antibodies, we performed a live virus assay using serum from individuals vaccinated with either the Pfizer (BNT162b2) or Moderna (mRNA-1273) vaccine. A prototypical virus (SARS-CoV-2/UT-HP095-1N/Human/2020/Tokyo; HP095 S-614G) with only the D614G substitution in its spike protein was used as a reference virus to compare with the neutralization titers against B.1.621. We selected 24 and 30 serum samples from individuals vaccinated twice with the Moderna or Pfizer vaccines, respectively. For both sets of samples, the serum was collected 7 to 32 days after the second dose.

**Fig. 1.**
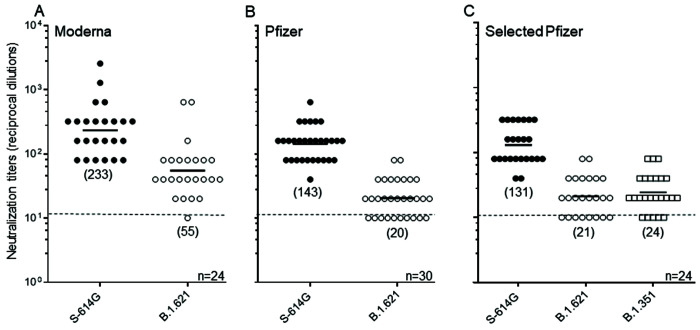
**Serum antibody responses to B.1.621 are reduced relative to the parental strain**. (**A and B**) Neutralization antibody titers were measured using human serum samples obtained from individuals vaccinated twice with either the Moderna (A) or Pfizer/BioNTech (B) vaccines. Neutralization assays were performed with an isolate of SARS-CoV-2 with only the D614G mutation in the spike (S-614G) or B.1.621. (**C**) Neutralization antibody titers were determined using a selected group of human serum samples obtained from individuals vaccinated twice with the Pfizer vaccine (Selected Pfizer). Neutralization assays were performed with an isolate of SARS-CoV-2 with only the D614G mutation in the spike (S-614G), B.1.621, or B.1.351 (beta), a variant of concern. In all groups, each circle or square represents an individual serum sample. The geometric mean of the neutralization titers obtained from a single experiment for each set is indicated by the solid black bars and the numeric values of the geometric means are indicated in parentheses. The lower limit of detection was a dilution of serum at 1:20 and is indicated by the dashed lines.

When compared against the reference virus (HP095 S-614G), we observed a 4.2-fold decrease in neutralization titers against B.1.621 with serum samples obtained from individuals vaccinated with the Moderna vaccine (Fig. 1A) and a 7.2-fold decrease with serum samples obtained from individuals vaccinated with the Pfizer vaccine (Fig. 1B). To compare this reduction in antibody sensitivity of B.1.621 against a variant of concern, we randomly selected 24 samples based on serum availability from individuals vaccinated with the Pfizer vaccine and performed the neutralization assay again, but with B.1.351 [Beta variant; antigenically most different from the prototype virus among the variants of concern prior to the emergence of the Omicron variant (*15*, *16*)]. In this assay, we observed a 6.2-fold decrease in neutralization titers against B.1.621 compared with the reference virus (HP095 S-614G), and a similar decrease in neutralization titers (5.5-fold) against B.1.351 (Fig. 1C).

To examine the durability of the antibody response against B.1.621, we performed neutralization assays with serum samples collected 6 months after the second vaccination with the Moderna or Pfizer vaccines. We observed similar reductions in neutralizing antibody titers compared to the earlier (7 to 32 days) timepoint after the second vaccination: a 3.4-fold decrease in neutralization titers against B.1.621 with serum obtained from individuals vaccinated with the Moderna vaccine (fig. S5A) and a 6.5-fold decrease with serum obtained from individuals vaccinated with the Pfizer vaccine (fig. S5B). Collectively, these findings suggest that B.1.621 has reduced sensitivity to antibodies elicited by vaccination with mRNA vaccines, similar to the reduction observed with the variant of concern B.1.351 (*17*, *18*).

### B.1.621 challenge of hamsters previously infected with SARS-CoV-2

To determine whether a previous infection with SARS-CoV-2 would protect against a re-challenge with B.1.621, we used a hamster model of SARS-CoV-2 infection (*19*, *20*). Hamsters (females; 5–6 weeks old, n=8) were first infected by intranasal inoculation with 10^3^ pfu of SARS-CoV-2 USA-WA1/2020 (WA-1), an early isolate with an aspartic acid (D) at amino acid position 614 of the spike protein (WA-1 S-614D). Six weeks after infection, serum was collected and used to determine neutralizing antibody titers against WA-1 S-614D and B.1.621. All infected hamsters had detectable neutralizing antibodies against WA-1, demonstrating seroconversion after infection (fig. S6). When B.1.621 was used in the neutralization assay with the same hamster serum, there was a 5.8-fold decrease in the neutralizing antibody titers compared with WA-1 S-614D (fig. S6), similar to the fold-decrease observed in neutralization titers with serum from vaccinated humans (Fig. 1 and fig. S5).

**Fig. 2.**
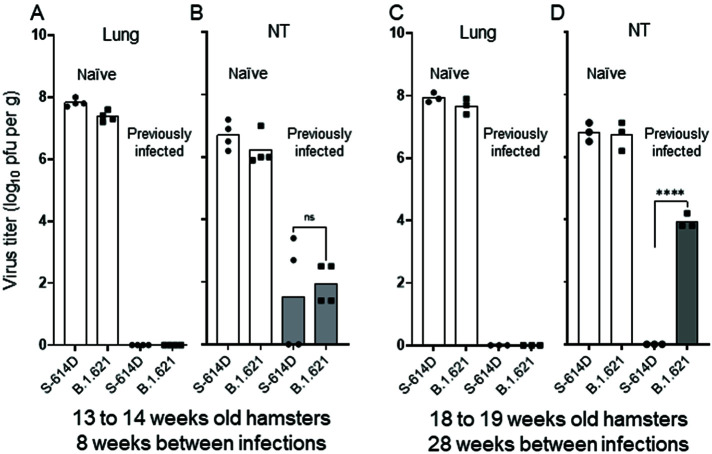
**Hamsters previously infected with WA-1 S-614D were partially protected from rechallenge with B.1.621**. (**A to D**) Virus replication in the lungs or nasal turbinates (NT) was measured in groups of naïve hamsters (open bars) or hamsters previously infected with WA-1 S-614D (gray bars). Hamsters were re-challenged with 10^3^ pfu of S-614D (circles) or B.1.621 (squares) at 8 weeks (A and B) or 28 weeks (C and D) after the first infection. Symbols in the figure indicate individual hamsters in each group from a single experiment (n=3 to 4 per group). Statistical significance was determined in the previously infected hamsters by a Student’s *t* test between the two challenge viruses (S-614D and B.1.621). Symbols on the x-axis represent virus titers below the limit of detection (10 pfu/g). ****p<0.0001; ns, not significant.

We next examined whether the reduction in reactivity of antibodies induced upon WA-1 S-614D infection would allow B.1.621 to replicate in hamsters previously infected with WA-1 S-614D. At 8 or 28 weeks after the primary infection, previously infected hamsters were re-challenged intranasally with 10^3^ pfu of WA-1 S-614D (prior infection: 8 weeks, n=4 or 28 weeks, n=3) or B.1.621 (prior infection: 8 weeks, n=4 or 28 weeks, n=3). Naïve, age-matched hamsters (13 to 14 weeks old; n=4 or 18 to 19 weeks old; n=3 for each virus) were also infected with either virus at the same dose. Three days after re-challenge, both WA-1 S-614D and B.1.621 replicated to high titers in the lungs and nasal turbinates of the naïve 13 to 14 weeks old (Fig. 2, A and B; open bars) and 18 to 19 weeks old hamsters (Fig. 2, C and D; open bars) with no difference in titers. In contrast, neither isolate was recovered from the lungs of either group of previously infected hamsters (Fig. 2, A and C). In the nasal turbinates, WA-1 S-614D and B.1.621 re-challenge led to similar titers in hamsters that were initially infected 8 weeks prior (Fig. 2B, gray bars). In contrast, B.1.621 grew to significantly (*p*<0.0001) higher titers compared to WA-1 S-614D in the animals that were initially infected 28 weeks prior (Fig. 2D, gray bars).

### Serum transfer studies in hamsters infected with B.1.621

With protection afforded against B.1.621 in previously infected hamsters, we next assessed the protection of hamsters from infection provided by administration of human convalescent serum. Hamsters (n=3 per group) were infected with 10^3^ pfu of WA-1 S-614D or B.1.621. Twenty-four hours after infection, each group of hamsters was treated through intraperitoneal injection with human serum obtained after: 1) vaccination with two doses of the Pfizer vaccine (vaccinated only group); 2) infection with SARS-CoV-2 (A.1 lineage), then vaccination with two doses of the Pfizer vaccine (infection-vaccinated group); or 3) administration of pre-pandemic, pooled control serum (control group). Four days after infection, virus titers were assessed in lung samples.

**Fig. 3.**
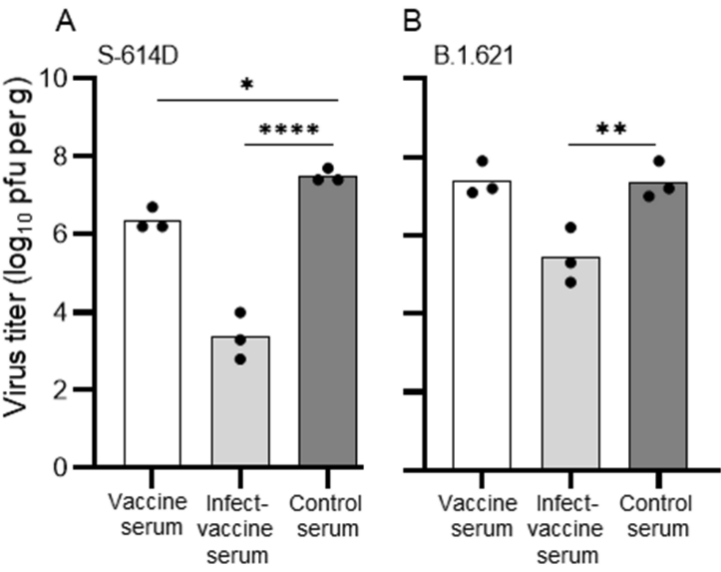
**SARS-CoV-2 titers are decreased in the lungs of infected hamsters after passive transfer of convalescent human serum**. Virus replication of WA-1 S-614G (A) or B.1.621 (B) on day 4 after infection with 10^3^ pfu of either virus in groups of hamsters treated with convalescent human serum obtained after: 1) vaccination with two doses of the Pfizer vaccine (vaccine serum); 2) infection with SARS-CoV-2 (A.1 lineage), then vaccination with two doses of the Pfizer vaccine (infect-vaccine serum); or 3) administration of pre-pandemic, pooled control serum (control serum). Symbols in the figure indicate individual hamsters (n=3 per group) from a single experiment. Statistical significance was determined by an ordinary one-way analysis of variance (ANOVA) test followed by Tukey’s multiple comparisons, where control serum was the control group. *p <0.01, **p <0.001, ****p <0.0001.

After infection with WA-1 S-614D, there was reduction in viral titers in the lungs of hamsters in the vaccinated only and infection-vaccinated groups (10- and 10,000-fold reduction, respectively) compared to the control group (Fig. 3A). In contrast, serum treatment was less protective against B.1.621 infection with only a 100-fold reduction in the lungs of hamsters in the infection-vaccination group compared to the control group (Fig. 3B).

## DISCUSSION

The SARS-CoV-2 B.1.621 (Mu) variant was designated as a variant of interest by the WHO because the constellation of mutations in its viral genome, particularly in the gene encoding the spike protein, may indicate immune escape from antibodies elicited by vaccines. Here, we demonstrated that this variant shows reduced reactivity to serum samples from individuals vaccinated twice with either the Moderna or Pfizer vaccine, a reduction in antibody reactivity similar to that of B.1.351, a variant of concern. We observed similar reactivity to antibodies with the B.1.621 variant when we tested hamster convalescent serum from animals previously infected with an early isolate of SARS-CoV-2. Despite this reduction in neutralization titers with convalescent hamster serum, previously infected hamsters that were re-challenged with the B.1.621 variant had no detectable replicating virus in the lungs and limited virus replication in the nasal turbinates, most likely due to limited immunity in the mucosal tissues. Human convalescent serum treatment of hamsters infected with B.1.621 was less protective compared to infection with an early S-614D isolate. Also in the hamster model, our data suggests that in the short-term, a previous SARS-CoV-2 infection will protect against a re-infection with the B.1.621 variant.

Our study has limitations. First, we examined the immune escape of B.1.621 by using serum samples from individuals vaccinated with the Pfizer/BioNTech or Moderna mRNA vaccine. It is possible that results may differ depending on the vaccine platform; therefore, serum samples from individuals vaccinated with different types of vaccines (such as protein-based or inactivated vaccines) should be explored for their neutralization capability against B.1.621, sub-lineages of B.1.621, and other emerging variants. In addition, our study was limited in the amount of time between the primary infection and re-challenge (28 weeks); therefore, the long-term protection afforded by a previous infection or vaccination against B.1.621 is unknown. Also, although hamsters are a useful model to study SARS-CoV-2 infection, it is difficult to recapitulate the genetic and SARS-CoV-2-specific immune diversity of humans in animal models. Such genetic and immune factors in humans may influence the overall outcome of infection by different SARS-CoV-2 variants in terms of virus replication, immune escape, and pathogenicity.

## MATERIALS AND METHODS

### Study design

This study was designed to characterize the SARS-CoV-2 B.1.621 (Mu) variant and determine whether mutations in its spike protein render it capable of immune escape. All experiments with SARS-CoV-2 were performed under biosafety level-3 agriculture (BSL-3 Ag) containment at the University of Wisconsin-Madison in laboratory space approved by the Centers for Disease Control and Prevention and the US Department of Agriculture. We investigated the neutralization of human serum collected from individuals under approved human study protocols. As part of the Immunity Associated with SARS-CoV-2 (IASO) study under the Institutional Review Board (IRB)-approved protocol at the University of Michigan Medical School (protocol number HUM00184533), blood samples were collected after signed informed consent was obtained from individuals who received the BNT162b2 (Pfizer-BioNTech) or mRNA-1273 (Moderna) mRNA vaccines. Human convalescent serum was obtained under a protocol approved by the Research Ethics Review Committee of the Institute of Medical Science, the University of Tokyo (approval number 2019-71-0201); blood samples were collected after signed informed consent was obtained.

We investigated virus replication in naïve, previously infected hamsters, and hamsters treated with human convalescent serum. All animal studies were performed after approval from the Animal Care and Use Committee at the University of Wisconsin-Madison in accordance with the approved protocol (protocol number V006426). The number of animals used in each experiment was chosen based on our previous studies with SARS-CoV-2 infection of animals in which the sample size was sufficient to evaluate a statistically significant difference between groups. The viruses in this study were sequenced to ensure they contained the correct amino acid substitutions in the spike protein, and the sequence of the B.1.621 variant was deposited in GISAID (EPI_ISL_1827552) with additional data deposited in the National Center for Biotechnology Information BioProject database (PRJNA752133; SRA accession numbers SRR15346232 and SRR15346231; biosample accession numbers SAMN20584285 and SAMN20584286). The cells used in this study were verified to be negative for mycoplasma contamination by monthly polymerase chain reaction (PCR) analysis. For all experiments, no data were excluded.

### Cells, viruses, and plaque assay

Vero E6-TMPRSS2 cells from the Japanese Collection of Research Bioresources (JCRB) Cell Bank (1819) were propagated in Dulbecco’s modified eagle medium (DMEM) containing 10% fetal bovine serum, antibiotics, and 1 mg/ml geneticin (G418) at 37°C with 5% CO_2_. WA-1 S-614D (USA-WA1/2020), HP095 S-614G (SARS-CoV-2/UT-HP095-1N/Human/2020/Tokyo), B.1.351 (hCoV-19/USA/MD-HP01542/2021), and B.1.621 (hCoV-19/USA/WI-UW-4340/2021) were propagated on Vero E6-TMPRSS2 cells. Virus titers in respiratory tissue samples (nasal turbinate and lung) were determined by performing plaque assays on Vero E6-TMPRSS2 cells. For lung samples, a piece of each lobe was collected and the pieces were pooled. After being frozen at -80°C for at least 24 hours, the tissue samples were homogenized in 1 ml of DMEM and clarified by centrifugation. Undiluted and a 10-fold dilution series of the clarified tissue samples (100 μl per well) were used to infect a monolayer of Vero E6/TMPRSS2 cells for 30 min at 37°C. The cells were then washed once to remove unbound virus and then overlayed with 1% methylcellulose media for four days. Crystal violet solution was added directly to the wells overnight to fix and visualize the plaques. Titration of the pooled lung lobe samples was performed in duplicate.

### Mouse and hamster studies

Male C57/BL6J mice (12 weeks old, strain 000664, Jackson Laboratories) or female Syrian hamsters (initially 4 to 5 weeks old, 8902F, Envigo) were used in these studies at the ages indicated in the text. While under isoflurane anesthesia, mice were intranasally infected with 10^4^ pfu of virus and hamsters were infected 10^3^ pfu of virus; both animal models received 50 μl of inoculum. In passive transfer studies, hamsters were first infected with 10^3^ pfu of the indicated virus isolate. Twenty-four hours later, the hamsters were injected intraperitoneally with human serum (1 ml per hamster). Health evaluations were performed daily. Tissues were collected at the indicated timepoints after the animals were humanely euthanized.

### Virus neutralization assay

Virus neutralization assays were performed with different isolates of SARS-CoV-2 on Vero E6-TMPRSS2. Serum samples (heat-inactivated at 56°C for at least 30 min) was diluted two-fold for a final concentration, after the addition of an equal amount of virus, of 1:20 to 1:10,240. After the virus (approximately 100 pfu) and diluted serum mixture was incubated at 37°C for 30 min, the mixture was added to confluent Vero E6-TMPRSS2 cells that had been plated at 30,000 cells per well the day prior in 96-well plates. The cells were then incubated for an additional 3 days at 37°C. Virus neutralization titers were determined as the highest serum dilution that completely prevented cytopathic effects.

### Statistical analysis

The sample sizes for the mice and hamster studies were determined from previous studies that demonstrated significant differences among groups. The researchers were not blinded to the group allocations during the experiments. Virus titers from animals are expressed as scatter plots with bars and individual datapoints, obtained by using GraphPad Prism 9. Neutralization titers and are displayed as scatter plots with individual datapoints for each serum sample. The geometric means are shown by the lines in each graph and the numeric values are in parentheses. An unpaired Student’s *t* test was performed between two experimental groups, whereas an ordinary one-way analysis of variance (ANOVA) test followed by Tukey’s multiple comparisons was performed between three experiment groups with one group being the control group.

## Supplementary Materials

### Other Supplementary Material for this manuscript includes the following:

MDAR Reproducibility Checklist
