# Supplementary material for: Characterization of the SARS-CoV-2 B.1.621 (Mu) variant
Source: Sci Transl Med. 2022 May 17:eabm4908. doi: 10.1126/scitranslmed.abm4908 (PMC9392899; doi:10.1126/scitranslmed.abm4908)
Supplement: Supplementary file 1 — Figs. S1 to S6 [file scitranslmed.abm4908_sm.pdf]

Science Translational Medicine
AAAS

Supplementary Materials for

Characterization of the SARS-CoV-2 B.1.621 (Mu) variant

Peter J. Halfmann *et al.*

Corresponding authors: Yoshihiro Kawaoka, yoshihiro.kawaoka@wisc.edu; Peter J. Halfmann, pjhalfma@wisc.edu

DOI: 10.1126/scitranslmed.abm4908

The PDF file includes:

Figs. S1 to S6

Other Supplementary Material for this manuscript includes the following:

MDAR Reproducibility Checklist
Data file S1

Supplementary Figures

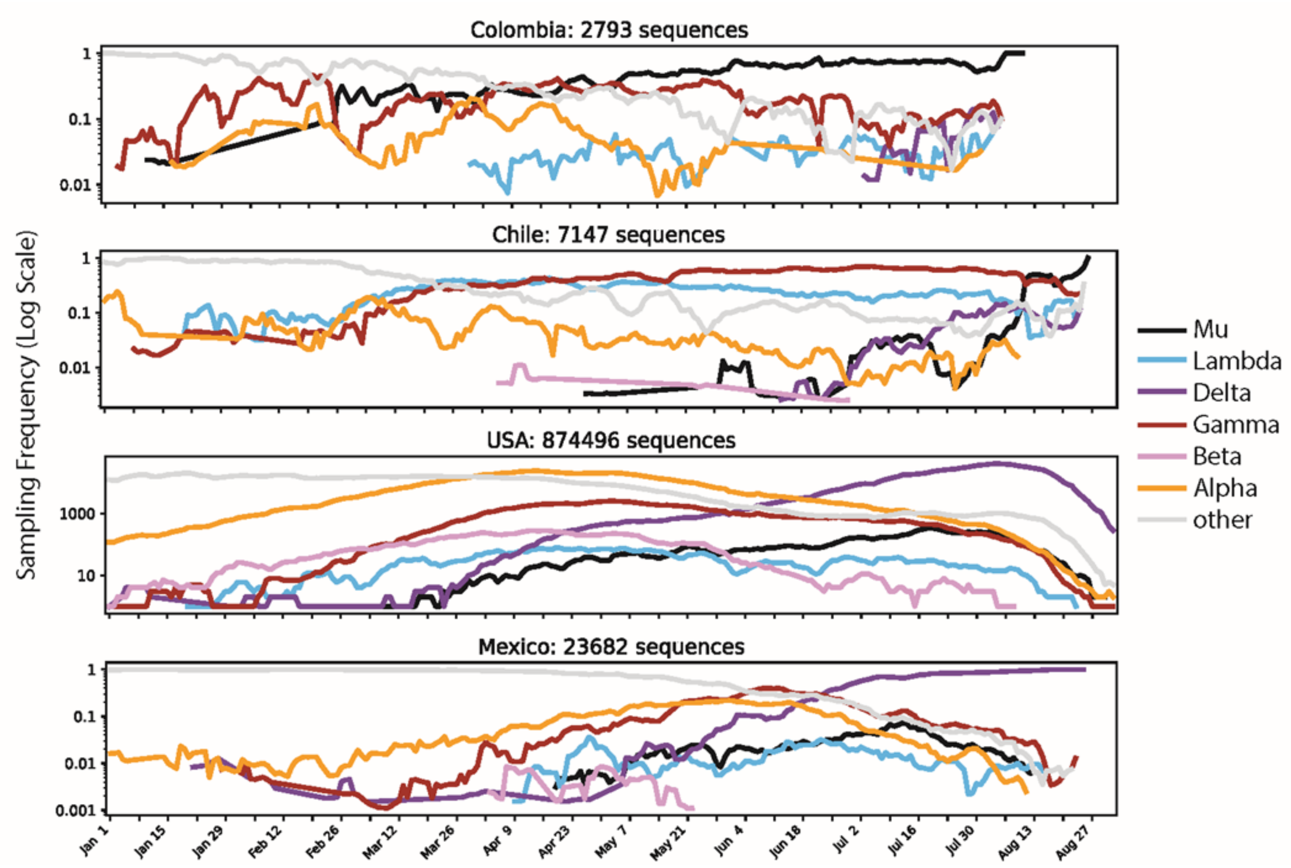

Fig. S1. Prevalence of B.1.621 in the Americas. Fractions of sampled variants, averaged by week and based on Pango lineage assignments of all sequences as provided by the Global Initiative on Sharing Avian Influenza Data (GISAID) database, were acquired using a September 3, 2021 data retrieval of GISAID data to the cov.lanl.gov analysis site (*4*). The following World Health Organization (WHO) variants of concern are highlighted: Alpha (B.1.1.7), Beta (B.1.351), Gamma (P.1), and Delta (B.1.617.2 plus all AY sub-lineages). We also include Lambda (C.37), a WHO variant of interest that is common in Latin America, and Mu (B.1.621). To simplify visualization, all other variants are grouped and their combined frequency is indicated by the gray line. The indicated number of sequence samples from January 1, 2021 to September 1, 2021 in Colombia, Chile, the United States (USA), and Mexico are shown, as examples of nations where the B.1.621 (Mu) variant was commonly sampled.

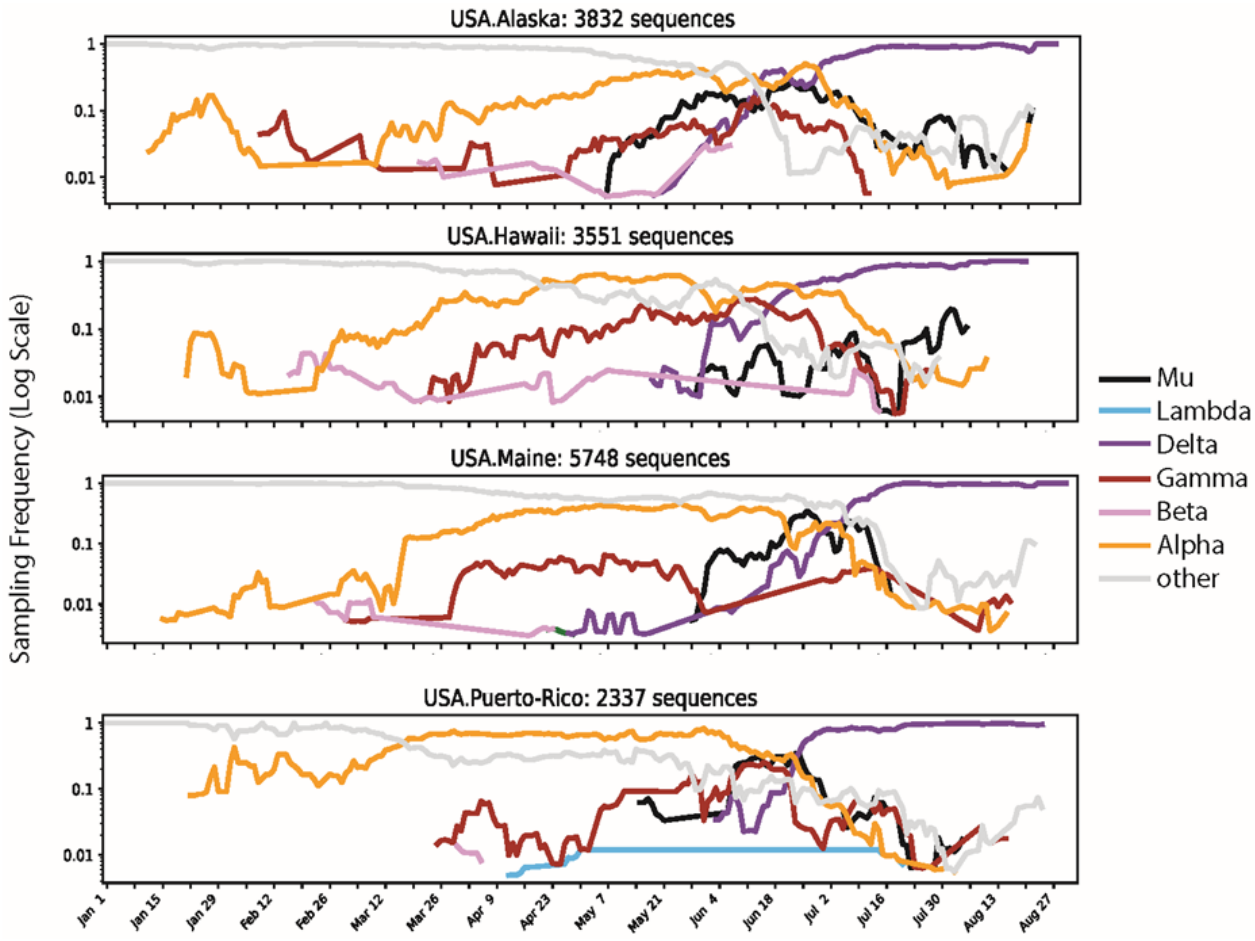

Fig. S2. Prevalence of B.1.621 in certain states or territories in the United States. Similar to fig. S1, the fraction of sampled variants is displayed, averaged by week, based on Pango lineage assignments of all sequences as provided by GISAID. Here, we highlight examples within the United States where the B.1621 (Mu) variant reached relatively high frequencies during the summer of 2021, including Alaska, Hawaii, Maine, and Puerto Rico.

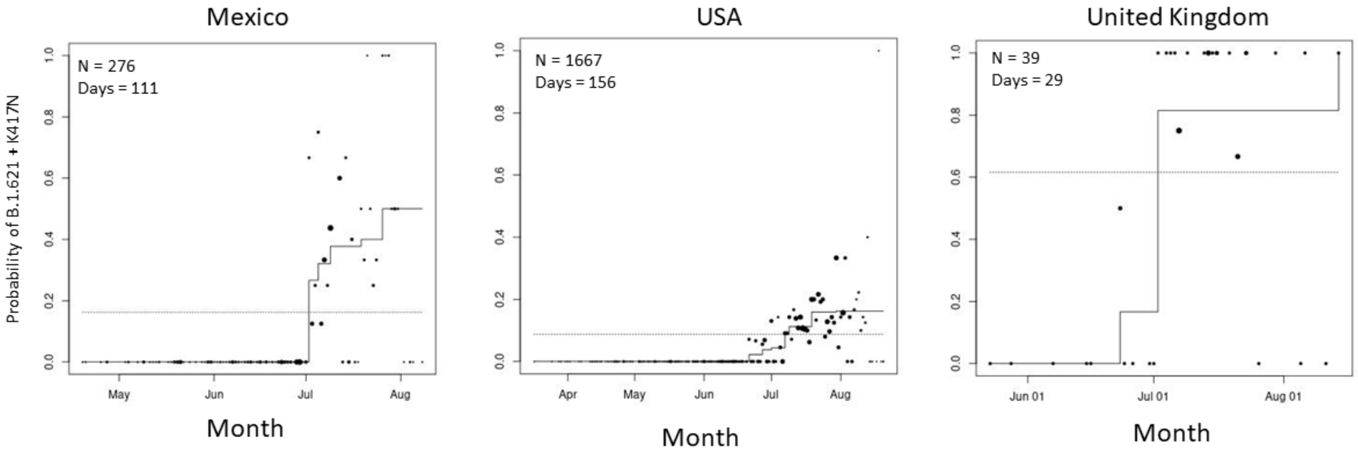

Fig. S3. Prevalence of B.1.621+K417N. To determine the frequency of B.1.621+K417N, we first identified all the nations where this subvariant has been found at least ten times, namely Mexico, the United States (USA), and the United Kingdom. We then used isotonic regression as described in Korber *et al.* to determine whether the subvariant was being sampled with an increasing frequency relative to other members of the B.1.621 lineage (*4*). Each black dot in the plot represents a day on which B.1.621 was sampled in the indicated county. The size of the dot reflects the sampling frequency of B.1.621, and the probability of N shows the probability that the spike of B.1.621 carries the K417N substitution. The dotted line is the fraction of the variant over the considered time window. N is the total number of B.1.621 viruses sampled, days indicates the number of days over which B.1.621 sequences were sampled, and the y-axis shows the proportion of the sample that was B.1.621+K417N relative to the total number of B.1.621 viruses sampled.

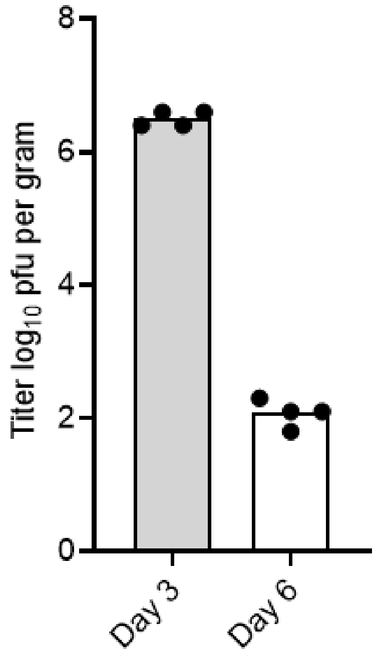

Fig. S4. C57BL/6J mice can be infected with the B.1.621 (Mu) variant of SARS-CoV-2. Virus replication in the lungs of mice was quantified on Days 3 and 6 after infection (n=4 mice per group). PFU, plaque forming units.

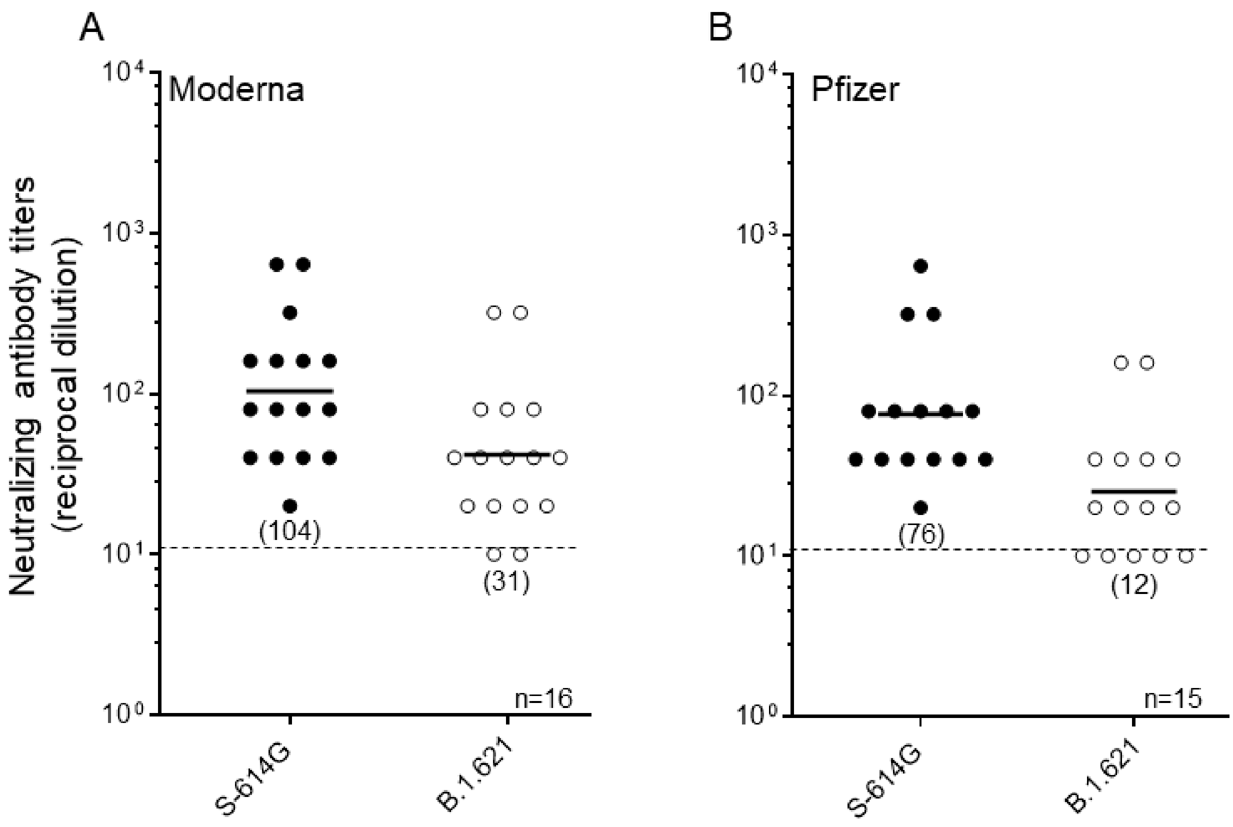

Fig. S5. Human serum antibody responses to B.1.621. Neutralization antibody titers were quantified using human serum samples obtained 6 months after the second vaccination from individuals vaccinated with either the (**A**) Moderna or (**B**) Pfizer SARS-CoV-2 mRNA vaccines. Neutralization assays were performed with an isolate of SARS-CoV-2 with only the D614G mutation in the spike (S-614G) or B.1.621. In all groups, each dot represents an individual serum sample. The geometric mean of the neutralization titers is indicated by the solid black bars and the numeric values are indicated in parentheses. The lower limit of detection was a serum dilution of 1:20, and is indicated by the dashed lines.

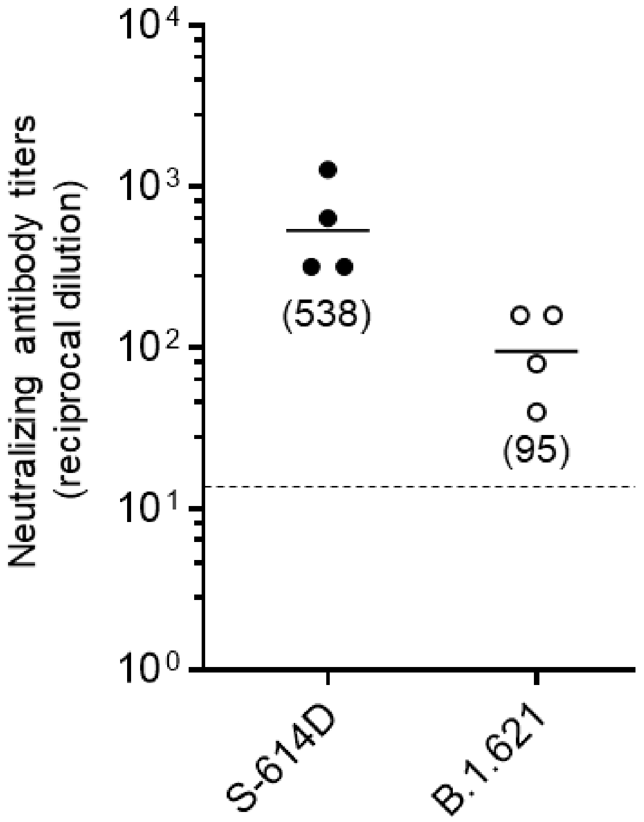

Fig. S6. Hamster serum antibody responses to B.1.621. Neutralization antibody titers were quantified using serum samples obtained from hamsters previously infected with WA-1, an isolate of SARS-CoV-2 with only the D614G mutation in the spike (S-614G). Neutralization assays were performed with WA-1 or B.1.621. Each dot represents a hamster serum sample (n=4 per group). The geometric mean of the neutralization titers is indicated by the solid black bars and the numeric values are indicated in parentheses. The lower limit of detection was a dilution of serum at 1:20.
